# Additional Remarks upon Infanticide

**Published:** 1833-05

**Authors:** Alfred S. Taylor

**Affiliations:** Lecturer on Medical Jurisprudence and Chemistry in Guy's Hospital.


### INFANTICIDE.

*Additional Remarks upon Infanticide.* By ALFRED S. TAYLOR, Esq., Lecturer on Medical Jurisprudence and Chemistry in Guy's Hospital. (Continued from p. 15.)

AT the conclusion of the last paper upon the subject, it was remarked that, among the objections urged by Dr. HUNTER against the employment of the hydrostatic test in cases of suspected infanticide, was the following; the air, to which the lungs owe their buoyancy, may be contained within the natural air-vesicles; and yet it may have resulted not from the act of respiration having been performed, but from the circumstance of attempts having been made to resuscitate the child, if found in a state of asphyxia, by inflating them artificially.

ESCHENBACH, and some other medical jurists, have considered that the supposition of an attempt of this nature being made by the mother is altogether inadmissible; but this, perhaps, is taking an extreme view of the case; for it is equal to assuming that every female who conceals the birth of her child, and is accused of having destroyed it, is really the murderer of her offspring. It certainly is improbable that a female, immediately after delivery, should possess either strength of body or presence of mind sufficient to perform this experiment; and it is obvious that, unless the inflation be properly conducted, *i. e.* unless the external orifices of the nostrils be closed, and a certain pressure be at the same time exerted, by forcing the larynx against the œsophagus, it will be much more likely that the stomach should be inflated than the lungs. But, setting aside the improbability which the assumption carries with it, we will suppose that a woman is secretly delivered of a stillborn child; that she attempts to restore it to life by blowing air into its mouth; that her efforts to restore it prove ineffectual; and that she secretly disposes of the body of the child, in order to remove all ground for suspicion.

The body of the child, being found, is given over for inspection to a medical practitioner; and he is required to state, at the inquest, whether the child had been born alive; *i. e.* whether it had respired after having been delivered. He finds the body altogether free from putrefaction,* the viscera of the thorax perfectly healthy, the lungs distended, projecting forwards, crepitating to the finger, slightly overlapping the pericardium, and presenting on their surface irregular patches of a pinkish-red colour. On placing them in water, he finds that they readily float, even when divided into smaller portions; although it is not improbable that some of these portions will be found to sink, since it is almost impossible, even by long-continued efforts at inflation, to distend these organs with air equally throughout their whole structure.

He has now, then, to determine whether the buoyancy of the portions of lung which float be owing to air introduced naturally or artificially; and this will lead him at once to put in practice those means which have been suggested to obviate the difficulty contained in this objection.

1st. If the experiment of inflating the lungs be performed upon these organs when removed from the thorax, the results will be more complete than where the inflation is performed while they are still contained within their proper cavity. It will be found, then, upon the first introduction of air, that they suddenly undergo a remarkable alteration of colour in certain situations only. I have generally observed that the apices and anterior margins have been the first parts to present these changes: they became of a bright pinkish-red colour, and in that way contrast strongly with the uninflated portions of the organs, which retain the black livid hue peculiar to the lungs of the stillborn child. By continuing the inflation, they may be made to acquire this bright colour over the whole of their surface; but it is rare that, on making a

* It is here assumed that the body is free from all signs of putrefaction, in order to make the question less complex. Where the lungs are not putrefied, it must necessarily follow that the air contained within their structure has been introduced either *artificially* by inflation, or *naturally* by the process of respiration.

section into their interior, we find an equal uniformity of colour. Judging from my own observations, I should be inclined to think that it would be impossible to produce this appearance in the lungs of a stillborn child, while retained within the cavity of the thorax; and therefore under the circumstances upon which the "objection" is raised. In all the experiments which I have performed upon the lungs of such subjects, when removed from the body, I have never failed to observe, even where considerable force was used in inflating the organs, and the operation was long continued, that their internal structure had become very unequally acted on by the air introduced, although their external surface appeared tolerably uniform in colour. I have found, upon making a section, that their substance was more or less mottled; that small patches of a livid colour, corresponding to parts not acted on by the air, intervened between the streaks of bright-red. This result is one for which we may be prepared, when we consider the manner in which the air is introduced. It has to overcome, in this case, the inertia of the dense mass into which it is propelled; and hence it will find its way only into those parts where it meets with the least resistance.

In the case of natural respiration, the phenomena are widely different:* the lungs are uniform in colour, whether we look at the surface, or make a section through their substance. The air, in fact, is equally diffused throughout their whole structure; a phenomenon which is readily explicable when we consider that the inertia of the lungs is in this case overcome by the action of the respiratory muscles, and that the air enters as into a species of vacuum. If these observations should be borne out by the results obtained in future investigations, it will follow that the examiner may determine, by a careful inspection of the organs, whether the air, to which the lungs owe their buoyancy, has resulted from artificial inflation, or from the act of respiration. But there are other means left open to us, by which we may corroborate our conclusions respecting the source from which the air has been derived.

2d. The principles of the test recommended by PLOUCQUET have been already explained. It has been remarked, that the effect of respiration is not only to render the lungs buoyant in water, but to increase their absolute weight. This results from the altered course of the blood; the whole of that

* I here presume, that the process has been fully and perfectly performed, and that the lungs are free from all morbid changes.

fluid contained in the ramifications of the pulmonary artery augmenting the weight of the organs. Ploucquet established a proportion, both for fœtuses which had respired and for others which had not; but the observations of CHAUSSIER and SCHMILT have tended, in the opinion of many, to invalidate his conclusions. It is certain that very varying results have been obtained; but it may be observed, that in the experiments sufficient attention does not appear to have been paid to two points: 1st, to the age of the fœtus; and 2dly, to the degree to which respiration has taken place. No inferences should be drawn either for or against the accuracy of this test, unless the experiments which have led to them have been performed upon fœtuses advanced to the same period of gestation. There may be variations in development at different ages, which may probably have given rise to the extreme variations obtained by experimentalists. It is admitted that there may be a difference in the degree of development of the body in fœtuses of the same age; but it is more than probable that this has far less influence on the results obtained by the test of Ploucquet, than the degree to which respiration has advanced. In many children, the function is but slowly and gradually established; and, therefore, in these it is not to be expected that the pulmonary circulation should be complete. In a few children which have died almost immediately after birth, where they have only lived to make one or two inspirations, it has been found that the circulation through the pulmonary vessels was by no means perfectly established. In both of these cases, the results afforded by Ploucquet's test must deviate from the average; and the deviation will be great, in proportion to the imperfect manner in which the process of respiration has been carried on.

I will here, however, suppose that the lungs are completely distended with air, and are universally crepitating to the feel. We apply the test of Ploucquet, and find that we obtain a proportion of about 1 to 60.* Our conclusion then will be, that the air has not resulted from respiration, but that it has been introduced artificially. In the latter case, of course, there cannot possibly be any augmentation of weight; since this augmentation in every instance is owing entirely to the altered course of the circulation; and thus, as in the case of the

* The proportion of 1 : 60 in the weight of the lungs and body before respiration, appears to be nearer to the truth than the proportion given by Ploucquet. This I have deduced as the mean of numerous observations made upon stillborn fœtuses arrived at the full period. After respiration, the proportion may be taken as 1 : 38.

"objection" from putrefaction, we have a means of determining the accuracy of our previous conclusion.

3dly. It was proposed by BECLARD, some years since, to try the effect of compression in cases of a suspected nature. He stated that where the air was introduced artificially, *i. e.* by inflation, it might be readily expelled by simple compression of the organs. This experiment I have frequently had occasion to perform; and in no case have I been unsuccessful in my attempts at expelling the air, even where the inflation had been carried to the most extreme degree. The experiment may be best performed by enclosing the lung, either entire or divided into four or five pieces, in cloth, and twisting the ends of the cloth in opposite directions. A moderate degree of force will suffice to expel the air, and, on placing the lung in water, after the experiment, it will be found to sink. I have almost invariably observed on these occasions, that from being of a pinkish-red colour, it passed to the dark livid hue which it had prior to inflation; and that it is by no means necessary to exert a degree of pressure sufficient to lacerate its structure, or disorganize it, in order to effect the entire expulsion of the air.

If we treat a healthy lung, which has fully undergone the process of respiration, in this manner, we shall find that the results will be widely different. No pressure will, so far as I have seen, suffice to expel the air, if derived from respiration. A portion may be expelled; but, so long as the interstitial structure of the lungs remains uninjured, sufficient will always be retained to preserve its buoyancy. In this simple experiment, we have then a very certain means of determining whether the buoyancy of the lungs be owing to the presence of respired air, or not.

But it may be urged, it is possible that respiration may have been so imperfectly conducted as that respired air may be expelled by the same degree of compression. This objection, however, has more plausibility than truth, as the following case, which is of recent occurrence, will prove. In the first communication which I made to this Journal on the subject of Infanticide, I represented myself as doubting what might be the result of compression upon lungs which had but imperfectly undergone respiration. I had not then succeeded in getting a fair case from which I could draw any legitimate conclusion respecting this point; but I flatter myself that the following, which presented itself to me by accident, will be considered as such.

On the 16th March, I examined the body of a child, which it had been found necessary to destroy in order to effect delivery. It lived, however, a sufficient time after the protrusion of its head to cry loudly, and therefore to exert its respiratory muscles.

The general appearance of the body shewed that the child had attained to the full period of gestation. On opening the thorax, the lungs were found projecting slightly forward over the pericardium, of a light-reddish colour, but giving no sense of crepitation to the finger. They had the external physical characters which these organs are known to have in children which have respired after birth; but the absence of all crepitation proved that the respiratory act could not have been perfectly performed. The colour of the external surface of the organs was perfectly uniform; a condition which I have never witnessed in lungs artificially inflated, except in those cases where the inflation had been carried to its most extreme degree, and where, therefore, a distinct feeling of crepitus invariably existed.

The lungs, being now removed from the thorax, were placed in a vessel of water, with the heart attached; and it was observed that they floated freely, immediately on a level with the fluid. The heart was now detached; and it was remarked, on separating the lungs by dividing the bronchi, that each lung floated equally well. A section was now made through the right lung, when it was found that the colour was uniform throughout; that the minute vesicles of air could not be perceived; and it was only by compressing the divided surface that any could be forced out. The right lung was then divided into sixteen pieces; and four of these pieces were forcibly compressed in a folded cloth; but it was found impossible to expel the air sufficiently to make them sink. They were divided into still smaller pieces, and again violently compressed with a force sufficient to rupture the cloth through several folds; but, on placing them in water, they still floated. They were again subdivided, when it was found that the air which they had hitherto retained was expelled; they therefore sunk in water, but the texture of the organ was entirely destroyed; and, indeed, was reduced to that state to which the healthy lung of an adult person would have been reduced by repeated hammering.

Upon subjecting the left lung entire to violent compression, it was found impossible to expel the air so as to cause it to sink.

Ploucquet's test could not in this case lead to any correct inferences, since the greater part of the cerebrum had been evacuated.

Now here, then, we have probably as striking an example of the effect of imperfect respiration on the lungs of a full-grown child as could be well conceived; for the child could not, at the utmost, have made more than one or two inspirations, according to the testimony of the accoucheur; its death was almost contemporaneous with its birth. Let us imagine, for a moment, that this had been a doubtful case; one in which it was required to know whether the air contained in the lungs had been derived from the act of respiration, or whether it had proceeded from attempts at artificial inflation. The uniformity of colour observed not only upon the surface of the organs, but throughout their whole substance, as well as the impossibility of expelling the air by compression, without absolutely reducing the divided portions to the state of pulp, are conditions which, to my mind, would have afforded the most satisfactory evidence that the child had performed the act of respiration. If we derive our conclusions from the compression of either lung, while entire, instead of dividing it into separate portions, the evidence will undoubtedly be still more satisfactory, at least, so far as my own observations extend; for, allowing the organ to be healthy and fully distended, in no instance have I failed in expelling the air by compression, where it had been artificially introduced; and, on the other hand, in no instance has it been possible for me to expel it, where it had resulted from the establishment of the process of respiration.

It may be, perhaps, considered by some that the above inferences have been too hastily drawn, and involve questions too important to be thus hastily dismissed; but the results are open to correction, and my great object will have been attained if I should succeed in drawing the attention of any member of the profession to this subject, and thereby lead them to the performance of experiments, in order to confirm or invalidate the results at which I have arrived; but, in the meantime, I may be allowed to conclude, with regard to this objection to the employment of the hydrostatic test, that,

1st. Where the lungs are crepitating to the finger, and uniformly of a pinkish-red colour, both superficially and internally, the air-vesicles being at the same time invisible;

2d. Where the test of Ploucquet gives the ratio of the weight of the lungs to the weight of the body as about 1 : 35;

3dly. Where the most violent and repeated compression is insufficient to expel the air from the organ, or to cause it to sink in water;

We have the strongest evidence that the air contained within the pulmonary cells has been introduced by the act of respiration: and, on the other hand, that,

1st. Where the colour of the lungs is not uniform throughout the mass, where dark livid patches are surrounded by bright red streaks, unequally distributed;

2d. Where the test of Ploucquet gives the ratio of about 1 to 60, in comparing the weight of the lungs and of the body; and

3d. Where, by moderate compression, we are able to expel the air, to cause the organ to assume a uniformly livid colour, and at the same time to deprive it of its buoyancy in water;

We have the strongest reason to conclude that the air which was contained within their structure had been introduced by artificial inflation.

In pursuing the subject, we find the following statement made by Dr. Hunter:

" If the child makes but one gasp, and instantly dies, the lungs will swim in water, as readily as if it had breathed longer, and had then been strangled."*

The question as to whether the child had been strangled or not, has nothing to do with the swimming of its lungs in water; because those who advocate the employment of the hydrostatic test, in cases of infanticide, do not look upon the floating of the lungs as any indication of the child's having been murdered, although Dr. H. invariably unites the two questions. We may therefore direct our attention merely to the first part of his proposition.

That a healthy full-grown child may, by one or two inspirations, so distend its lungs with air as to render them freely buoyant in water, is undoubted: the case which was just now cited for another purpose, may be taken as a clear proof of the fact. But, nevertheless, it would seem that, in some instances, a considerable time is required in order that the lungs should become perfectly dilated by the act of respiration. This has been more particularly observed in regard to children born before the full term, and in those which have been exposed to cold and inanition. In one instance, however, I have met with a case where, even after the lapse of six months from the time of birth, the lungs had not become fully inflated.

It was the case of a child, aged six months, which was supposed to have died by suffocation. Upon opening the thorax, the viscera were found perfectly healthy, but the whole of the inferior lobe of the right lung was, so far as

* On the Uncertainty of the Signs of Murder, &c., p. 33.

regarded colour, density, and structure, precisely like the lungs of the fœtus. No air had ever penetrated into it, and the line of demarcation by which it was separated from the lobe adjoining it precisely corresponded to the line of separation between the two. It had become developed in size, and there was no appearance of disease. When the whole of the lung was placed in water it floated; but, when the inferior lobe was separated from the others, it immediately sunk to the bottom of the vessel.

I shall hereafter have occasion to revert to this condition of the lungs, occasionally met with in newly-born children; but, granting the proposition made by Dr. Hunter, that "if the child makes but one gasp, and dies," its lungs will swim in water as readily as if it had lived longer, it remains to be inquired how this is to affect the application of the "test" in cases of infanticide.

The reader will at once perceive that it has no reference whatever to the question at issue. The point to be determined by it is, whether the child has respired or not after its birth; for the act of respiration is taken by the law as synonymous with life. If this be proved, and it be further shewn that the child has been really strangled by its mother, or any other person, it imports but little, in the eye of the law, whether the child has made but *one gasp*, or more, prior to its having been murdered; for it is not necessary that the child should respire for a certain period, in order that its destruction should amount to murder, or, otherwise, trials for infanticide would never be heard of in a court of justice; and the application of the hydrostatic test, as well as of every other test, might be very properly abandoned!

Dr. Hunter further observes, that "a child will very commonly breathe as soon as its mouth is born, or protruded from the mother, and in that case may lose its life before its body be born; especially when there happens to be a considerable interval of time between what we may call the birth of the child's head and the protrusion of the body: and, if this may happen where the best assistance is at hand, it is still more likely to happen where there is none."

The fact alleged by Dr. Hunter relative to the establishment of respiration before the child is perfectly born, cannot be disputed. It is a matter of daily experience; but a child can rarely perish under these circumstancess, unless accidentally suffocated by the clothes, or intentionally destroyed. Indeed, it has been well observed, that the fact of the establishment of the process of respiration in the child is sufficient to guarantee it from the influence of the ordinary causes of death, by which children, during birth, are liable to be cut off; such as interrupted circulation, by pressure on the cord, &c. But, granting that the child may breathe while in this position, and die prior to the completion of delivery, the application of the hydrostatic test will indicate that it has respired; but certainly it will not indicate whether the act of respiration has taken place prior or subsequent to its complete birth. This must ever remain a question of presumption; and it is well for the constitution of society that the crime of infanticide is not made to rest upon proofs of this fact.

The buoyancy of the lungs will here, as in every other case, prove that the child has breathed; but it will not prove that it has been murdered. Let us suppose, however, that there are evident marks of manual violence about a child, and that there are strong proofs that the mother has really murdered it. Let us also imagine that the hydrostatic test shews most clearly that the child has performed the act of respiration, we may naturally stop to inquire whether the law insists upon proof of the act having been performed after its complete birth, in order that the mother should be proceeded against upon the charge of infanticide. This is the substance of the difficulty involved in Dr. Hunter's proposition; a difficulty which does not in the least operate against the results obtained by the employment of the hydrostatic test, but affects only the interpretation put by the law upon the term "infanticide."

We may look in vain into the works of our chief legal authorities for a correct definition of the crime of infanticide. It is commonly called "child-murder;" but the exact period at which the offspring is considered to become a child is not stated. It would appear, from the ordinary proceedings on trials for infanticide, that the complete birth of the child, and its entire separation from the mother, should be proved before the charge could be maintained; or, otherwise, how are we to understand why counsel should almost invariably ask the medical witness whether a child might not breathe and die prior to its birth? as if this had anything more to do with the question of its wilful destruction, than where the witness was expressly required to state whether a child might not breathe and die after its birth? Let us suppose that a case occurs where the head of the child presents at the outlet, and the murderer destroys it by thrusting an instrument into its brain before it can possibly have respired, are we to look upon this as a crime or not? It certainly does not come within the legal definition of criminal abortion; and it is almost equally certain that the murderer would escape a conviction, if tried for infanticide. The law considers that the child has not lived unless it be proved to have respired, although, morally and physiologically speaking, an individual who destroys a child under these circumstances is as truly a murderer as one who destroys it after it has lived several days. Let us suppose, however, that the destruction of the child is not attempted until its head has escaped from the outlet, and until it has performed the act of respiration. In such a case, the medical witness might prove that the child had enjoyed life, and that it had been wilfully destroyed; but is it possible to imagine that the prisoner will be acquitted of the charge, unless the witness can distinctly swear whether the child had respired prior or subsequent to its separation from the mother? It would be a libel upon the common principles of justice to affirm it, since, if such a rule were rigidly insisted on, no conviction could ever take place in those cases where the child is destroyed soon after its birth!

The term "infanticide" ought to be applied to the destruction of the life of a child, either during or after its birth, and whether the act of respiration has been performed or not. If the law insist upon some proof of the child having respired, before it will allow of its having lived, the hydrostatic test may be usefully applied by the witness. If, however, it should require proof of the complete birth and separation of the child from its mother, even where respiration has clearly taken place, before it will admit of the charge of infanticide, it is requiring more than medical testimony can afford, and more than either reason or justice can demand.

Let us, on the other hand, allow to Dr. Hunter the inference which is to be drawn from his proposition, and let us admit that, in a clear case of infanticide, a counsel is justified by law in demanding of the medical witness, whether a child might not respire, and yet not be born alive, in order to exculpate the prisoner from a charge of murder; it must necessarily follow, at least in many instances, that there will be a want of evidence of the fact, in which case the accused party, however great the guilt or enormity of the crime, must be acquitted. In such a case, it will be evident that the escape of the accused cannot be attributed to any defect in the application of the hydrostatic test, or in the medical testimony generally, but to a kind of special pleading, tolerated in the law as an evil necessarily accompanying the dispensation of a vast proportion of good!

Some have gone farther than Dr. Hunter, and have stated that a child might breathe while its *head was in the uterus;* and others have said, that they have heard the child cry while in this situation, explaining the fact by stating that the mouth of the child presented at the dilated orifice of that organ. Most of these cases have about the same degree of authenticity as those recorded by LIVY the historian, of children being heard to laugh and weep before birth.* Some modern physiologists consider it as proved, that a child may respire while its head is still in the uterus, and that it may die prior to the completion of delivery; but, without admitting it as proved, we may allow it as possible; in which case, the same remarks are applicable as those already made upon the circumstance of respiration taking place while the head is at the outlet, and the body remains undelivered.

Some opponents to the hydrostatic test have stated that a child may be born, and live for a short period, and yet not respire; in which case, the results of experiments on the lungs would lead to the inference that it had been born dead. I have already, in a previous paper, referred to instances of this kind, of which two are recorded by WRISBERG, where the children were born enclosed in the membranes. In one of these, according to his statement, life was prolonged for seven minutes without respiration, and in the other for nine minutes. Whether we admit these cases of Wrisberg or not, we may admit the possibility of a child being born alive without respiring; at least, for the sake of seeing how far it can operate as an objection to the main subject of our inquiry. Now the application of the hydrostatic test can only inform us whether life, *in the legal sense of the term*, has existed in the offspring, *i. e.* whether respiration has taken place or not; and it is upon the *legal* and not upon the *physiological* acceptation of the term *life* that the whole of our inquiries respecting its utility must be based. It is, then, from a want of proper reflection upon the subject that such an objection has ever been raised to the employment of the hydrostatic test. It is rather to be considered as a censure upon the state of our law, which calls for a particular proof of vitality in *every* case; when life may be present, and yet such proof cannot be afforded. The act of respiration affords unequivocal evidence of the child having lived; but it is absurd to suppose that it is thereby inferred that a child may not, physiologically speaking, enjoy life without it. If the law will not hold an offender who murders a child during birth, and before respiration is established, to be guilty of murder, it is not probable

* It is mentioned by this historian, that in one instance the child, while in the uterus, was heard to utter the words "*io triumphe!*" Vide MAHON, *Mèdicine Legale*, tome ii.

that it will hold an individual to be guilty of infanticide who destroys the child *after* its birth and before it has respired. It has been considered, perhaps, as more conducive to the interests of humanity to regard every child which has not respired as stillborn; hence the circumstance of life existing in the offspring without the establishment of respiration, although physiologically certain, does not appear to be legally admissible.

Those who regard this as an objection to the use of the docimasia pulmonaris, may be called upon to bear in mind that an individual may die from the effects of poison, while, by our tests, we may be unable to discover the smallest trace of poison within his body after death! Would it not, however, be considered unreasonable, if a person were to contend that, because our means of research did not serve us in this case, we should therefore abandon the use of tests altogether; yet such is the very drift of this species of argument, whether the question be one of poisoning or of infanticide!

Again, it has been asserted that a child may be born alive, and respire; but, owing to the act of respiration having been feeble, or owing to the presence of disease in the organs, the lungs may not contain sufficient air to render them buoyant in water. We may therefore fall into the error of pronouncing a child which was born living to have been stillborn.

The influence thus attributed to disease is perhaps more imaginary than real, and may be dismissed in a very few words. The practitioner must, of course, be presumed to be able to distinguish between a healthy and a diseased state of the fœtal lungs. Now, it is not probable that disease will so affect the whole of the lungs as to render every part specifically heavier than water: hence, if, upon their being divided into pieces and placed in water, it be observed that some portions float, he will have to satisfy himself respecting the origin of the air which they contained, and may draw his inferences accordingly. If the sinking of some portions be owing, as supposed, to the effects of disease, this will be readily determined by simple inspection; and thus, all the difficulties of the case will vanish. If the whole of the organs be diseased, and not a single portion be found to float, it is clear that no evidence can be derived from them in that particular case!

The lungs, it is said, may also sink in water where the child has respired, and where their structure is perfectly healthy; for it is contended, respiration being a process which is in many instances but slowly established, it may be some time before sufficient air becomes introduced to render them buoyant in water. Cases of this kind have been collected by

Hunter, Morgagni, Haller, and others. Schenk relates an instance where an infant lived four days, and cried several times, whose lungs sunk in water; but it is probable that, if the particulars of this case be true, there was great disease of the organs. It has already been stated that this imperfect dilatation of the organs by respiration is chiefly met with in children that come into the world prematurely; and it is not improbable, as Dr. Hutchinson has suggested, that exposure to cold and want of food may also have an influence in retarding the process. Cases of this kind must, however, be considered as exceptions to the general rule. In the instance just now related, of the child destroyed by the accoucheur in order to bring about its delivery, I found that, although it could not have made more than one or two inspirations, the lungs floated as freely as if it had lived for many days; and that the most forcible efforts were insufficient to expel the air which they contained.

It may suffice to state, with regard to this objection, that it may be always set aside by making our experiments on those portions of the lungs which contain air; and the inferences thus obtained will be as conclusive as if we had had the whole of the organs to experiment on. We know that such a condition of the lungs as that indicated may occasionally exist; and therefore we may readily explain, in such a case, why the whole of the divided portions of the organs do not float.

This, then, concludes the examination of the objections which have been brought by various writers to the employment of the hydrostatic test, in cases of infanticide; and it remains for the professional witness to determine if possible, by experiment, the degree of validity to be attached to the answers which have been made respectively to each.

He is called to examine the body of a child, and he finds that the lungs freely float in water. If the body be not in a state of putrefaction, the floating of the lungs will prove either that they have been artificially inflated, or that the child has respired. If then it be found that the air contained is readily expelled by compression, and the other signs be present already enumerated under the head of artificial inflation, we must conclude that the air has been artificially introduced. Should it be found impossible to expel the air by compression, we have the clearest evidence that it has resulted from respiration, which may be corroborated by the presence of other concomitant signs.

If, however, the lungs should be found to sink in water, even when divided into small pieces, and the substance of the organs be at the same time perfectly healthy, we have the strongest evidence that the child has not lived to respire. If any of the divided portions contain sufficient air to render them buoyant, we have only to determine, according to the rules above given, whence that air has proceeded.

By the hydrostatic test, therefore, we only attempt to solve one question, namely, whether the child has respired or not; and it is a most serious mistake to suppose that the life of a woman depends upon the result of its application. The proofs of her guilt, if accused of having destroyed her off-spring, will depend upon the concurrence and combination of many circumstances; and indeed, it is difficult to conceive of a case in which medical testimony would be alone sufficient for her conviction. The reverse, however, seems now to be the principle acted on. The medical witness has only to swear that the proofs of the child having lived (respired) after its birth are *unsatisfactory*; and the judge has only to declare that Dr. Hunter's opinion has settled the question relative to the employment of the hydrostatic test, when the accused, although there can be but little moral doubt of her guilt, is instantly acquitted.

It would appear invidious to cite cases in support of these statements, but the reports of every circuit will amply testify to their truth. How far such a state of things is reconcileable with the proper administration of justice, it is for the moral or political philosopher to determine; but he whose object is not, through a misplaced feeling of humanity, to shew favour to the guilty, but to uphold the authority of the law, cannot but regret that principles so mischievous should be tolerated, and that medical science should be the means by which they are enforced and perpetuated!

*35, Great Marlborough street; April 22*, 1833.

